# The Activity Screening of Hmong Herbs *Caesalpiniaminax* and an Antitumor Effect Study

**DOI:** 10.1155/2020/3585736

**Published:** 2020-10-26

**Authors:** Cong Huang, Minghong Dong, Jinfang Luo, Haibing Qian, Jingjie Zhang, Yongqi Huang

**Affiliations:** ^1^Basic Medical College, Guizhou University of Traditional Chinese Medicine, Guiyang 550025, Guian, China; ^2^Guizhou Province Key Laboratory of Prescription and Syndrome Pharmacology in Chinese Medicine, Guiyang 550025, Guian, China; ^3^School of Pharmacy, Guizhou University of Traditional Chinese Medicine, Guiyang 550025, Guian, China

## Abstract

**Aim:**

To screen active sites from roots, stems, and leaves of *Caesalpinia minax* and study its antitumor effect.

**Methods:**

Human liver cancer HuH-7 cells were used to screen the active sites of *C*. *minax* by MTT assay, and the polar extracts were analyzed by high-performance liquid chromatography (HPLC). The medicated serum prepared from rats was used to investigate the effect on the proliferation of Hepa 1–6 mouse hepatoma cells. The H22 hepatoma-bearing mice model was established and treated with the petroleum ether extract of stems and leaves (HY②) by intraperitoneal injection (20, 60 mg·kg^−1^) and intragastric (100, 300 mg·kg^−1^) for 12 days.

**Results:**

The petroleum ether extracts were the effective active site. The IC_50_ value of the petroleum ether extract of stems and leaves (HY②) was 58.9 *μ*g·ml^−1^, and that of the roots (HG②) was 46.79 *μ*g·ml^−1^. The grey relational analysis cleared that the 11 common peaks of the active extracts had a close correlation with the antitumor activity. The medicated serum prepared by ip. administration had a significant inhibitory effect on the Hepa1-6 cells, but it had no inhibitory effect by intragastric administration. High-dose administration significantly reduced tumor size in H22 hepatoma-bearing mice, and the tumor inhibition rates were 64.47% and 53.41%. Necrosis of tumor cells, infiltration of inflammatory cells, and fibrous tissue proliferation were promoted, and the expression of the proliferating cell nuclear antigen (PCNA) and vascular endothelial growth factor (VEGF) was reduced compared with the control group.

**Conclusion:**

The petroleum ether extract of *C*. *minax* had a significant antitumor activity. An immunohistochemical study showed that, through inhibiting the expression of the VEGF and growth of the tumor blood vessel, the proliferation of tumor cell and expression of PCNA can be inhibited.

## 1. Introduction

Malignant tumors have always been an important public health problem in China. For many years, the conservative treatment of malignant tumors has mainly focused on radiotherapy and chemotherapy, but they also have adverse reactions. It is worth mentioning that many traditional Chinese medicine and ethnic medicine have good effects for reducing adverse reactions when they exerted their therapeutic effects for tumor [1]. Besides, they can better promote the recovery of patients, improve symptoms, and increase the survival rates [2].

*Caesalpinia minax* Hance, belonging to the family Leguminosae, is also known as “Nan-she-le”. The seeds of this plant are named as “ku-shi-lian” in the south of China which is widely used as Hmong herbs medicine for the treatment of dysentery and hematuria [3], and it is also used in the treatment of swelling, exogenous fever, rheumatic pain, and traumatic injury [4]. The traditional medicinal part of *C*. *minax* was seeds [3]; previous phytochemical investigations showed that the main chemical constituents of this plant are flavonoids, steroids, and terpenoids [5–9], and its diterpenes had an inhibitory effect on the growth of AGS, HepG-2, and MCF-7 tumor cells [10, 11]. However, the antitumor effect of extracts from stems and leaves of *C*. *minax* is not yet reported. In this paper, active sites were screened from roots, stems, and leaves of *C*. *minax* by human liver cancer HuH-7 cells experiment (*in vitr*o), and then, its antitumor effect was studied based Hepa 1–6 mouse hepatoma cells and the H22 hepatoma-bearing mice model (*in vivo*). This study provided experimental evidence for expanding the range of medicinal use of *C*. *minax* and applying it to the clinical antitumor complementary treatment.

## 2. Materials and Methods

### 2.1. Plant Material

The plants of *Caesalpinia minax* Hance were collected from Ceheng County in Guizhou Province, China, and identified by professor He Shunzhi (Guizhou University of Traditional Chinese Medicine). A voucher specimen (GZTM 0018421) was deposited in the Guizhou University of Traditional Chinese Medicine, Guiyang, China.

Dried stems and leaves of *C*. *minax* (1.0 kg) were ground into a coarse powder and refluxed with 5000 ml of 75% ethanol for 3 times (each time refluxed for 4 h), and then, the extracts were combined and concentrated under pressure to obtain the ethanol extract (HY①) yielded 133.0 g. After the residue was dried, it was extracted 3 times with 12 times, 8 times, and 6 times of water through heating (separately refluxed for 2, 1, and 0.5 h), and the solvent was recovered to obtain the water extract (HY⑥) yielded 42.7 g. The ethanol extract was, then, dispersed in H_2_O and extracted with different organic solvents to clarify the obtained petroleum ether extract (HY②) yielded 21.3 g, ethyl acetate extract (HY③) yielded 11.3 g, n-butanol extract (HY④) yielded 20.0 g, and hydromethanolic extract (HY⑤) yielded 25.3 g. The abovementioned operation is repeated to extract the roots of *C*. *minax* (1.0 kg) to the obtain ethanol extract (HG①) yielded 81.0 g, petroleum ether extract (HG②) yielded 5.5 g, ethyl acetate extract (HG③) yielded 23.9 g, n-butanol extract (HG④) yielded 3.17 g, hydromethanolic extract (HG⑤) yielded 25.3 g, and water extract (HG⑥) yielded 13.3 g.

### 2.2. Drugs and Reagents

The medium (DMEM/HIGH GLUCOSE, batch No. NXKO731), fetal bovine serum (FBS, batch No. NXCO582), and trypsin (batch No. SH30042.01) were from HyClone, and the antibodies for PCNA and VEGF were purchased from DAKO Company (USA). Epirubicin was obtained from Beijing Botai High-tech Co., Ltd. Fluorouracil injection was purchased from Tianjin Jinyao Amino Acid Co., Ltd. First-grade edible rapeseed oil (batch No. 20131024) was purchased from Dadaoquan Chongqing Grain and Oil Co., Ltd. Paraformaldehyde tissue fixation solution was provided by the Department of Pathology, Affiliated Hospital of Guizhou Medical University. Phosphoric acid, dimethyl sulfoxide (DMSO, batch No. 2012062), acetonitrile (batch No. 20121018), and methanol (batch No. 20120201) were purchased from Tianjin Kemiou Chemical Reagent Co., Ltd (all of chromatographic purity). Antibodies against VEGF (clone VG1）and PCNA (clone VG1) were rat anti-human antibodies (DAKO North America, Inc.).

### 2.3. Animals

Rats (Wistar, male and female, weight 180–200 g, certificate No. SCXU (Yu) 2007–0005) and Kunming mice (male and female, weight 18–22 g, certificate No. SCXU (Yu) 2007–0005) were tested by the Third Military Medical University Provided by the Animal Center. Those were housed under a standard conditions breeding room (20–25°C; 60–75%; 12-h dark/light cycle) with free access to food and water. All experimentation were conducted according to the guidelines of the Committee for Ethics in Animal Research (CEPA), and the experimental procedures were approved by the Local Committee on Animal Care and Use.

### 2.4. Cell Line

The human liver cancer HuH-7 cell line and Hepa1-6 mouse hepatoma cell line were provided by the Shanghai Cell Bank of the Chinese Academy of Sciences. The H22 mouse hepatoma cell line was from the Kunming Institute of Zoology, Chinese Academy of Sciences. The cells were retained in Dulbecco's modified Eagle's medium (DMEM), which contained L-glutamine (2 mM), penicillin *G* (100 U ml^−1^), 10% heat-inactivated FBS, and streptomycin (100 mg·ml^−1^).

### 2.5. Assay for Human Liver Cancer HuH-7 Cells Proliferation with *C. minax* Extracts

Human liver cancer HuH-7 cells were used to screen the active sites from roots, stems, and leaves of *C*. *minax* by MTT assay. The IC_50_ value of the active sites on human liver cancer HuH-7 cells was measured.

#### 2.5.1. Screening of Antitumor Active Fractions (MTT)

The methods were described in previous papers [12, 13]; in brief, human liver cancer HuH-7 cells in logarithmic phase were collected and digestion, centrifugation, and counting. Then, a cell suspension with a concentration of about 1 × 10^5^ cells·ml^−1^ was prepared with a fresh medium containing 10% fetal bovine serum. First, 100 *μ*l cell suspension was added into each well in a 96-well plate and incubated in a CO_2_ incubator for 24 h. For the petroleum ether, ethyl acetate, and n-butanol extracts, DMSO was used to aid solubilization. The hydromethanolic and water extracts were dissolved with NS, then filtered through a 0.22 *μ*m microporous membrane, and diluted with the culture medium to 100 *μ*g·ml^−1^, 20 *μ*g·ml^−1^, and 4 *μ*g·ml^−1^. Besides, the culture medium (100 *μ*l) of medicated serum was added to each well of the administration groups, and 3 replicates were set for each group. The control group (with the culture medium, DMSO, or NS) and zeroing group (without HuH-7 cells) were set. Cells were reincubated for 48 h, the supernatant was discarded from the 96-well plate, and then, 50 *µ*l of 5 mg·ml^−1^ MTT serum-free medium solution was added to each well. After incubated for additional 4 h, the medium was removed and the DMSO (150 *µ*l) solvent was added to each well. The cells were shaken with a shaker for 10 min, and then, the absorbance was read at 570 nm with a Model 2S-3 Microplate Reader (Beijing). The experiment was repeated twice, and the inhibition rate (IR_1_) of cell proliferation was calculated. The samples of petroleum ether, ethyl acetate, and n-butanol were compared with the DMSO control group, and the samples of the hydromethanolic and water extracts were compared with the NS control group.

(1) $$\begin{array}{c} \text{IR}1=\frac{\left(\text{OD value of control group}-\text{OD value of experimental group}\right)}{\text{OD value of control group}}\times 100\%. \end{array}$$

#### 2.5.2. Antitumor Effect of Active Sites

According to the preliminary results of cell experiment screening, the petroleum ether extracts were effective active sites. The IC_50_ of HuH-7 cells was determined by the same MTT assay. For HY② and HG②, DMSO was used to aid solubilization, then filtered through a 0.22 *μ*m microporous filter, and then, diluted with the culture medium to 200, 100, 50, 25, and 12.5 *μ*g·ml^−1^. Besides, two control groups (with DMSO or PBS), five positive control groups (with epirubicin at concentrations of 20, 10, 5, 2.5, and 1.25 *μ*g·ml^−1^), and a zeroing group (without HuH-7 cells) were set. The experiment was repeated twice, the inhibition rate (IR_1_) was calculated, and the IC_50_ was calculated by software.

### 2.6. HPLC Analysis

The HPLC analysis was validated for precision, repeatability, and stability, and the relative standard deviation (RSD) was less than 3.0% in all cases, which meets the technical requirements for HPLC chromatogram.

#### 2.6.1. Preparation of the Sample Solution

Samples of each polar extracts (0.2 g) were accurately weighed and placed it in a 10 ml volumetric flask, methanol was added and made up to volume, shaken well, and sonicated for 30 min. It was taken out and cooled at room temperature and filtered through a microporous membrane (0.45 *µ*m) to obtain the test solution.

#### 2.6.2. Chromatographic Conditions

The extracts were performed with an Agilent 1100 series high-performance liquid chromatographic instrument (Agilent, quaternary pump, degasser, USA). The chromatographic column was a Dima column (250 mm × 4.6 mm, 5 *µ*m). The column temperature was 25°C. The detection wavelength was 240 nm. The eluent flow was 1.0 ml  min^−1^, and the injection volume was 10 *µ*l. A gradient elution was performed with mobile phases A (0.1% phosphoric acid), B (methanol), and C (acetonitrile) as follows: 0 min, 5% B, and 5% C; 1 min, 15% B, and 15% C; 60 min, 25% B, and 25% C; 140 min, 33% B, and 33% C; 270 min, 40% B, and 40% C; 340 min, 45% B, and 45% C; and 360 min, 50% B, and 50% C.

### 2.7. Assay for Hepa 1–6 Mouse Hepatoma Cells Proliferation with Medicated Serum

HY② was dissolved with edible rapeseed oil, and the rats were intraperitoneally injected to find the maximum tolerated amount of 0.4 g·kg^−1^ for 4 days, and the intragastric administration was used to find the dosage to be 2 g·kg^−1^.

Twelve rats were randomly divided into five groups, four administered groups (2 rats·group^−1^), and one control group (4 rats). According to the pretest results, the ip. group was administered at 0.4 g·kg^−1^ and the ig. group was administered at 2 g·kg^−1^. In the control group, the edible rapeseed oil was intraperitoneally injected and intragastrically at a dose of 1 ml·100 g^−1^. Administration was given twice a day (4-day), 30 minutes after the last administration, blood was taken from the femoral artery, and serum was prepared by conventional methods.

Hepa 1–6 mouse hepatoma cells with medicated serum was determined by the MTT assay. The medicated serum was diluted with nonmedicated serum to a concentration of 50%, 25%, and 12.5%. 100 *μ*l of the culture medium of medicated serum was added to each well of the administration groups, and 3 replicates were set for each group. The control group (with nonmedicated serum) and zeroing group (without Hepa1-6 cells) were set, and final serum concentration of each group was 50%. The experiment was repeated three times, and the inhibition rate (IR_1_) of cell proliferation was calculated.

### 2.8. H22 Hepatoma-Bearing Mice Experiment

The H22 hepatoma-bearing mice model was established and randomly divided into the control group, fluorouracil (FU) group, intraperitoneal injection (ip.) high-dose group, ip. low-dose group, intragastric (ig.) high-dose group, and ig. low-dose group. According to the acute toxicity test, the LD_50_ value of HY② was 1700 mg·kg^−1^. In the ip. group, 1/30 of LD_50_ was high-dose, 1/3 of high-dose was low-dose; in the ig. group, 1/6 of LD_50_ was high-dose, 1/3 of high-dose was low-dose. After 12-day continuous administration, the tumor weight was measured and assessed for pathological changes, and the expressions of the proliferating cell nuclear antigen (PCNA) and vascular endothelial growth factor (VEGF) in tumor tissues were measured.

#### 2.8.1. Modeling, Grouping, and Administration

The H22 mouse hepatoma cell line was resuscitated, and cells were seeded into the abdominal cavity of healthy mice to produce ascites tumor-derived mice (0.2 ml each mice). After 7 days of culture, the mice with ascites were disinfected in a sterile environment in a clean bench, and then, the nonbloody ascites was extracted with a injector and placed in a sterile container. After the ascites was diluted with normal saline, the cells were counted and the tumor cell concentration was adjusted to 1.0 × 10^7^ cells·ml^−1^. Then, the mice were subcutaneously inoculated with 0.2 ml each in the left anterior axillary, that is, the number of tumor cells per mouse was 2.0 × 10^6^. The abovementioned method for establishing the H22 hepatoma-bearing mice model has been described previously [14].

One day after inoculation of tumor cells, 60 mice with normal activity were randomly grouped (*n* = 10) divided as follows: the control group, FU group (83 mg·kg^−1^), ip. high-dose group (60 mg·kg^−1^), ip. low-dose group (20 mg·kg^−1^), ig. high-dose group (300 mg·kg^−1^), and ig. low-dose group (100 mg·kg^−1^). HY② was administered from day 3 after vaccination (at 0.2 ml·10 g^−1^). The control group, ip. and ig., was administered edible rapeseed oil of the same capacity. The control group and administration group were administered once a day, and the FU group was administered once every other day. Then, all mice were administered continuously for 12 days and sacrificed on the 13th day, and tumors were quickly and completely peeled off on ice and weighed to calculate the tumor inhibition rate (IR_2_).

(2) $$\begin{array}{c} \text{IR}2=\frac{\left(\text{weigh of tumors in the control group}-\text{weigh of tumors in the treatment group}\right)}{\text{weigh of tumors in the control group}}\times 100\%. \end{array}$$

#### 2.8.2. Assay for Tumor Tissues Morphology (HE)

The tumor tissue was cut into two sections and immediately fixed in 10% formalin for 24 hours and dehydrated and embedded in experimental steps according to conventional methods. Tumor tissue sections (4 *μ*m) were routinely stained with hematoxylin-eosin (HE), and the pathological changes were observed by using a light microscope. Tumor cell lesions were scored using tumor cell necrosis, inflammatory cell infiltration, and fibrous tissue proliferation as indicators of pathological changes (lesions occupy 1 point below 30%, 2 points between 30 and 50%, and 3 points above 50%).

#### 2.8.3. Assay for the Expression of the PCNA and VEGF in Tumor Tissues

The immunohistochemical method followed the instructions of the immunohistochemistry kit and reference [15]. The immunohistochemical experiments were grouped as follows: administration control group, fluorouracil (FU) group, intraperitoneal injection (ip.) high-dose group, ip. low-dose group, intragastric (ig.) high-dose group, and ig. low-dose group.

After 13 days of administration, the mice were sacrificed and the tumor tissues were quickly taken out on ice, cross sectioned into two pieces (4*μ*m-thick), soaked in 10% formalin for 24 hours, dehydrated according to conventional methods, and embedded. The tissues were deparaffinized and hydrated to make into 4 *μ*m thick sections, stained with the proliferating cell nuclear antigen (PCNA), rinsed with distilled water and PBS, soaked in the newly configured 0.3% H_2_O_2_ buffer (in 0.05 Tris-HCL buffer, pH = 7.6) for 30 minutes at room temperature, rinsed with distilled water, placed in PBS for 10 minutes, incubated in the primary antibody for 20 minutes at a constant temperature of 4°C, rinsed with PBS for 10 minutes, incubated with EnVision^TM^ for 20 minutes, rinsed with PBS for 10 minutes, then incubated with the color source substrate solution for 20 minutes, rinsed with distilled water, counterstained, and mounted on the slide. Brown-yellow particles appeared when the PCNA was positive, and PCNA-positive was located in the nucleus. Five tissues with 400-fold fields were randomly selected to observe and count the number of PCNA-positive tumor cells.

According to the abovementioned experimental steps, the tissues were made into two sections for immunohistochemical staining. Brown-yellow particles means VEGF-positive, and the VEGF-positive protein was located in the cytoplasm. The 5 dense areas of neovascularization in the tumor tissue were selected at low magnification (100-fold fields), then the number of microvessels in one field of view in each dense area was counted at 200-fold fields, and the average value of the number of microvessels in 5 different areas was used to represent the microvessel density of the tumor.

### 2.9. Statistical Methods

Statistical analysis was performed using SPSS 23.0 statistical software, and data were expressed as Mean ± SD (*n* ≥ 3); *P* < 0.05 was considered statistically significant.

## 3. Results

### 3.1. Effect of *C. minax* Extracts on Proliferation of HuH-7 Cell

According to the in vitro antitumor activity screening experiments on HuH-7 cells, the results showed that HY② and HG② had obvious inhibitory effects on HuH-7 cells (*P* < 0.05, *P* < 0.01), but no inhibitory effect on other extracts (Table 1). Therefore, the petroleum ether extracts of *C*. *minax* (HY② and HG②) were determined to be effective active sites. The average IC_50_ value of the two experiments that inhibited the growth of HuH-7 cells was taken as the IC_50_ value. The IC_50_ value of epirubicin was 4.7 *μ*g ml^−1^, HG② was 46.79 *μ*g ml^−1^, and HY② was 58.9 *μ*g ml^−1^, which suggested that the inhibitory effect on HuH-7 cells was HG② > HY② (Table 2).

### 3.2. HPLC Analysis

**Figure 1:**
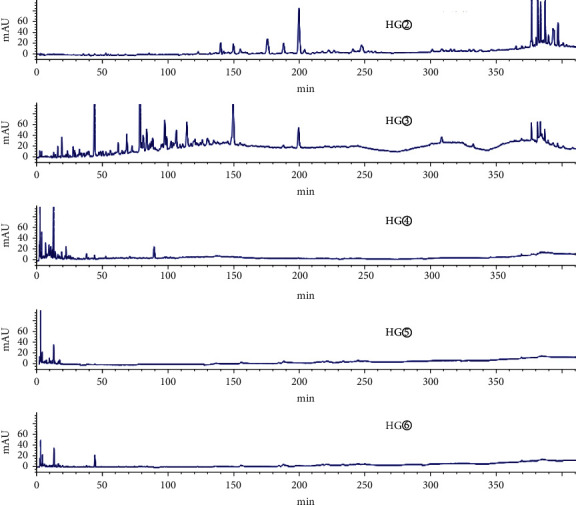
HPLC chromatogram of the petroleum ether extract (HG②), ethyl acetate extract (HG③), n-butanol extract (HG④), hydromethanolic extract (HG⑤), and water extract (HG⑥) from the roots of *C. minax*.

**Figure 2:**
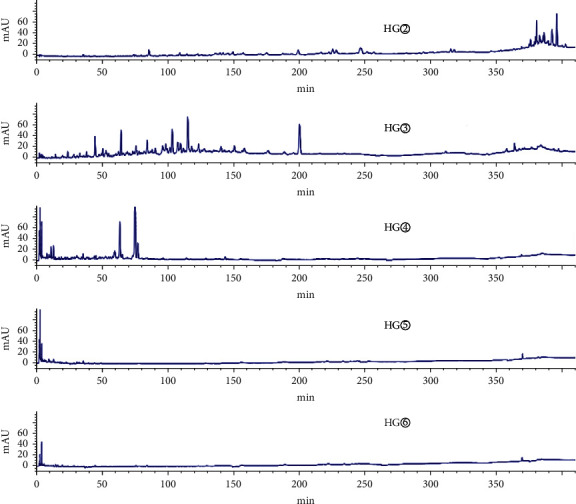
HPLC chromatogram of the petroleum ether extract (HY②), ethyl acetate extract (HY③), n-butanol extract (HY④), hydromethanolic extract (HY⑤), and water extract (HY⑥) from the stems and leaves of *C. minax*.

As shown in the HPLC chromatogram of the polar extracts from the roots of *C*. *minax* (Figure 1), compared with HG②, the three samples HG④, HG⑤, and HG⑥ had no chromatographic peaks between 100 min and 410 min, and most HG③chromatographic peaks were between 0 min and 150 min. It showed that the components of the stems and leaves were less than those of the roots. HY② was significantly different from HY③, HY④, HY⑤, and HY⑥ (Figure 2). HY② and the other four samples had no common peaks in this period.

**Figure 3:**
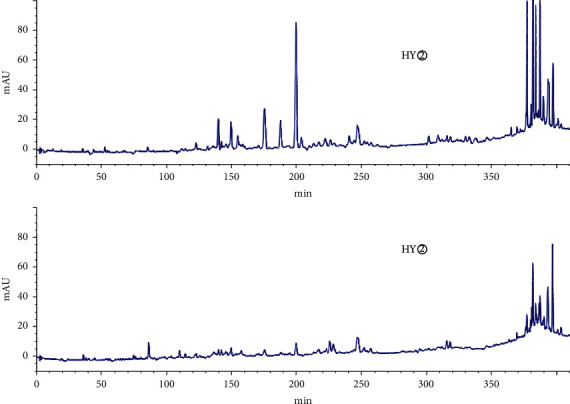
Comparison of HPLC chromatograms of petroleum ether extracts (HG② and HY②) from *C*. *minax*.

**Figure 4:**
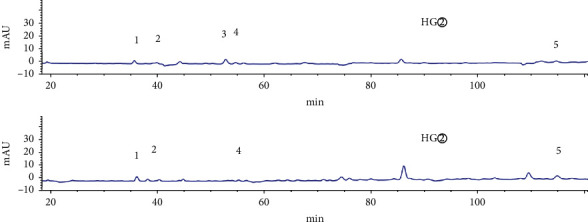
Common peaks 1–5 of petroleum ether extracts (HG② and HY②) from *C*. *minax*.

**Figure 5:**
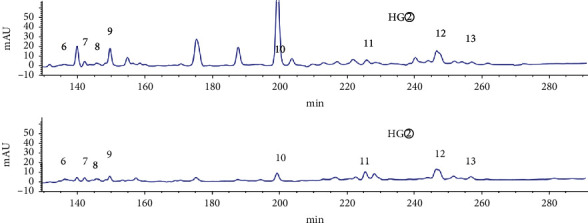
Common peaks 6–13 of petroleum ether extracts (HG② and HY②) from *C*. *minax*.

**Figure 6:**
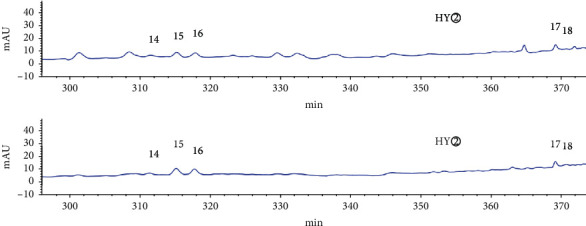
Common peaks 14–18 of petroleum ether extracts (HG② and HY②) from *C*. *minax*.

**Figure 7:**
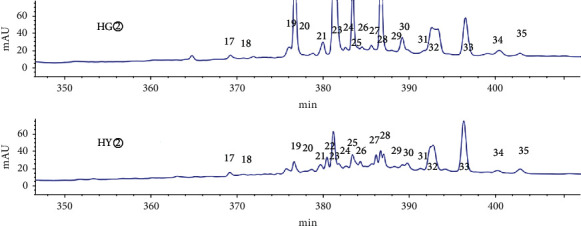
Common peaks 17–35 of petroleum ether extracts (HG② and HY②) from *C*. *minax*.

HPLC analysis found that active sites (HG② and HY②) had 35 common peaks (Figures 3–7), and the total peak areas of common peaks were 45659.7 and 21683.9. The total number of peak areas of HG② is more than twice that of HY②.

### 3.3. Grey Relational Analysis of Common Peaks of Active Sites and Antitumor Activity

According to the preliminary screening results, the petroleum ether extracts of *C*. *minax* (HY② and HG②) were determined to be effective active sites. The IC_50_ value for the petroleum ether extracts effect on human liver cancer HuH-7 cell was used as a pharmacodynamic index for the active chemical composition, and the correlation between the common peak and the antitumor activity was analyzed by grey correlation [16–18]. It was concluded that the contribution of the chemical components represented by each peak to the effect varies. Also, the common peaks with a greater correlation to the efficacy (correlation coefficient > 0.8) were 24 > 35 > 7 > 4 > 30 > 17 > 32 > 29 > 12 > 21 > 13 peaks (Table 3).

### 3.4. Effect of Medicated Serum on Proliferation of Hepa 1–6 Mouse Hepatoma Cells

As shown in Table 4, with the increase of the concentration of the medicated serum prepared by ip. administration, the OD value gradually decreased. It showed that the proliferation inhibition rate depends on the drug concentration. High and medium concentration medicated serum prepared by ip. administration had a significant inhibition rate on Hepa 1–6 mouse hepatoma cells (*P* < 0.05) compared with the control group. The medicated serum prepared by ig. administration had no significant difference compared with the control group and has no inhibitory effect on tumor cells.

### 3.5. Analysis of Tumor Weight

As shown in Table 5, the FU group significantly reduced tumor weight (*P* < 0.001) compared with the control group; high dose of the ip. group significantly reduced tumor weight (*P* < 0.01); high dose of the ig. group also significantly reduced tumor weight (*P* < 0.05). It showed that high doses of ip. and ig. inhibited tumor growth significantly, but low doses of ip. and ig. had no significant effect on tumor growth.

### 3.6. Analysis of Tumor Tissue Pathological Scores (HE Staining)

**Figure 8:**
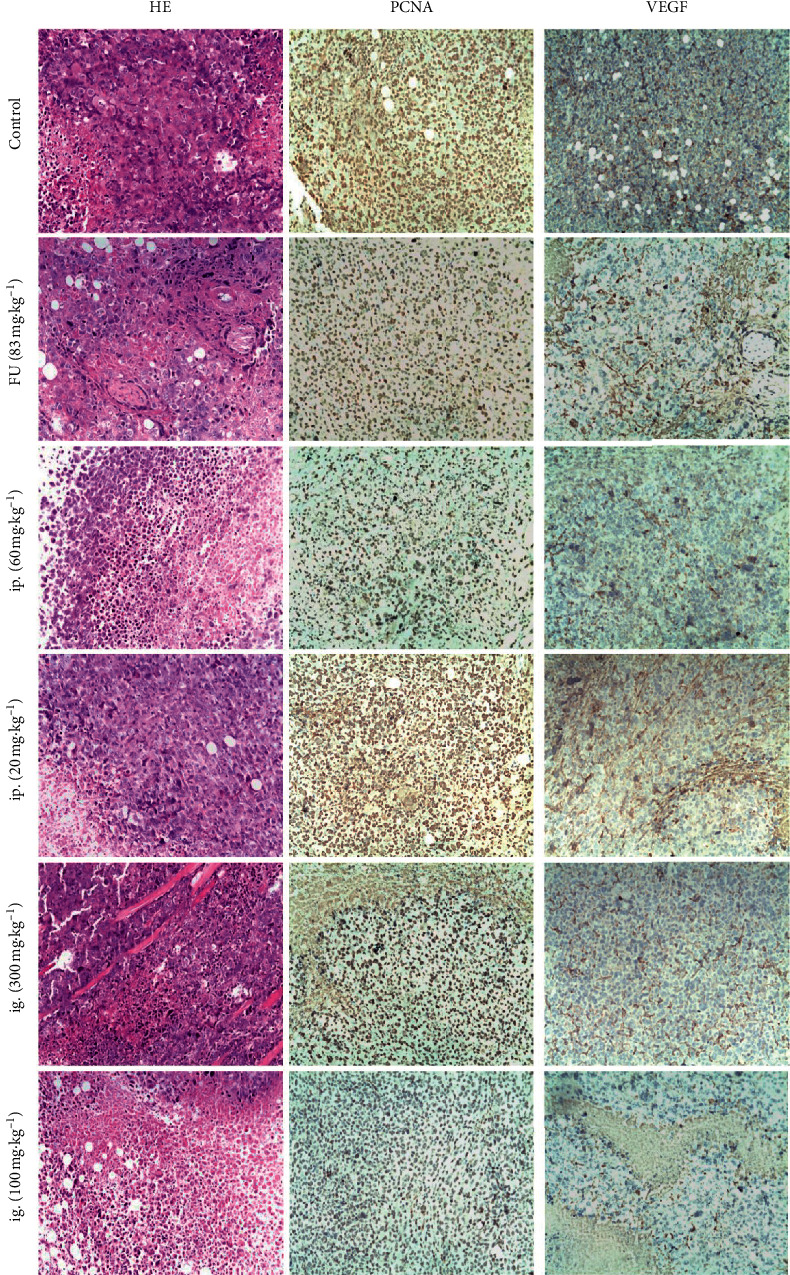
Effects of HY② on tumor tissue of H22 hepatoma-bearing mice. H22 hepatoma-bearing mice were treated with FU (83 mg·kg^−1^) and HY② by intraperitoneal injection (20, 60 mg·kg^−1^) and intragastric (100, 300 mg·kg^−1^) for 12 days. For HE, PCNA, and VEGF staining, light microscopy 200x was performed. Bars = 100 *μ*m.

As shown in Figure 8, in the control group, the tumor tissue cells had different sizes, irregular shapes, large nucleus, polymorphism, and deep chromatin staining. In other groups, tumor cells showed varying degrees of necrosis, inflammatory cells infiltration, and fibrous tissue proliferation. Compared with the control group, the tumor cells in the FU group and the high-dose groups were reduced significantly, and the tumor cell lesions score was significantly different (*P* < 0.05) (Table 6). But, the low-dose administration had no significant effect on tumor pathological changes.

### 3.7. Analysis of the PCNA Expression

The microscope results showed that PCNA-positive products were located in the nucleus (Figure 8). Compared with the control group, the expression of the PCNA in the high-dose groups was significantly lower (*P* < 0.05) (Table 6). FU injection, ip. and ig. high-dose administration, reduced the number of tumor cell proliferating cell nuclear antigens and inhibited tumor cell proliferation significantly. However, low-dose administration had no significant effect on the number of tumor cell proliferating cell nuclear antigens.

### 3.8. Analysis of the VEGF Expression

Under the microscope, VEGF-positive products were found in the cytoplasm. It was expressed in tumor tissue capillaries, small veins, and arterial endothelial cytoplasm (Figure 8). Compared with the control group, FU injection and high- or low-dose administration inhibited tumor tissue angiogenesis significantly (*P* < 0.001) (Table 6).

## 4. Discussion

In this study, the petroleum ether extracts of different parts of *C*. *minax* had significant inhibitory effects on HuH-7 cells, which were effective active sites. From the comparison of the HLPC chromatogram, it was known that the two contain similar chemical components, and the total number of peak areas of the petroleum ether extract in roots was more than twice that of stems and leaves. According to the results of the efficacy test, although its action intensity was greater than that of the stems and leaves, the actual medicinal effect and the total peak areas did not show a corresponding multiple, which indicated that the component peaks that exert the medicinal effect had different contributions. Moreover, the grey relational analysis was used to calculate the contribution of the chemical components represented by the peaks to the antitumor effect, and it was cleared that the 11 common peaks had a greater correlation with the antitumor activity. Obviously, these ingredients play a synergistic role, which was in line with the characteristics of the comprehensive effect of traditional Chinese medicine with “multicomponents and multitargets” [19]. At the same time, it was suggested that *C*. *minax* contained many antitumor compounds, which provided abundant resources for isolating lead compounds with antitumor activity in the next study.

When ip. administered at the maximum dose, the medicated serum of the petroleum ether extract of *C*. *minax* had an obvious inhibition on Hepa1-6 cells proliferation. But, the ig. administration is 5 times higher than ip., and its medicated serum had no obvious inhibitory effect on the proliferation of Hepa1-6 cells. It is suggested that the bioavailability of oral administration was low, and the reason needs to be further studied. In the H22 hepatoma-bearing mice model, tumor weight analysis and tumor tissue HE staining results showed that FU injection and high doses of the petroleum ether extract of *C*. *minax* could promote tumor cell apoptosis and necrosis significantly, thereby reducing tumor weight. However, low doses had no significant effect on tumor growth, which suggested certain dose dependence. ig. and ip administration had the same effect in antitumor, which are inconsistent with the results of in vitro experiments, and the reasons need to be further studied.

PCNA is a specific nucleic acid protein that exists in all phases of the proliferation cycle of normal and tumor cells [20]. Its expression level reflects the degree of cell proliferation. As the degree of damage to tumor cells increases, its expression rate increases significantly. The PCNA is a cofactor for DNA polymerase *δ*, and its content is periodic [21]. During the quiescent period of cell division, the content is very small. It starts to increase in the late G1 phase, peaks in the S phase, and decreases significantly in the G2 and *M* phases [22]. It can be seen that the change in the amount of PCNA is consistent with DNA synthesis and can be used as an index to evaluate the cell proliferation status [23]. In this study, the decreased expression of thee PCNA indicated that the petroleum ether extract of *C*. *minax* effectively reduced tumor cell DNA synthesis, thus resulting in the inhibition effect on tumor cell proliferation.

The growth and metastasis of tumors rely on new blood vessels to provide nutrients and excrete metabolites. Tumor angiogenesis is the formation of capillaries induced by tumor cells from the existing vascular network [24]. The VEGF is the strongest and most specific one among the currently known angiogenic factors [25]. By increasing vascular permeability, it strongly stimulates endothelial cell proliferation and promotes angiogenesis. The VEGF plays an important role in the proliferation and migration of vascular endothelial cells [26]. Immunohistochemistry found that the VEGF expression in the administration group was significantly lower than that in the FU group. This result indicated that the petroleum ether extract of *C*. *minax* reduced VEGF-induced signal transduction of vascular endothelial cells and inhibited tumor neovascularization.

Therefore, all the abovementioned results indicated that the petroleum ether extracts from the roots, stems, and leaves of *C*. *minax* contained a variety of antitumor ingredients and they had synergistic antitumor efficacy. They inhibited blood vessel growth of tumor through reducing the VEGF expression level in H22 hepatoma-bearing model mice and also inhibited tumor cell proliferation through reducing the PCNA expression. Multiple antitumor ingredients from *C*. *minax* inhibited tumor growth through multiple antitumor effects, and it provided abundant resources for the development of antitumor lead compounds in the future study. There are 6 species of medicinal plants of the genus *Caesalpinia* in Guizhou Province, one of which is *C*. *minax* which is rich in resources, and its seeds are widely used in folk medicine and have significant curative effects [27, 28]. This article, for the first time, studied the antitumor activity of the extracts isolated from roots, stems, and leaves of *C*. *minax* and also provided experimental evidence for *C*. *minax* clinic use. The purpose for this study is to expand the scope of medication of *C*. *minax* so that resources can be fully utilized in antitumor complementary treatment.

## 5. Conclusions

In this study, all the experimental results showed that petroleum ether extracts of the roots, stems, and leaves of *C*. *minax* effectively inhibited tumor proliferation for the first time. Therefore, the scope of extracts can be extended to roots, stems, and leaves so that the resources of *C*. *minax* can be fully utilized in antitumor complementary treatment. In the later stage, qualitative analysis will be performed on the common peaks of petroleum ether extracts from the roots, stems, and leaves of *C*. *minax*. It provided research foundation for the development of new antitumor Chinese patent medicine.

## Figures and Tables

**Table 1:** Effect of extracts from *C*. *minax* on HuH-7 cells proliferation (*n* = 6, Mean ± SD).

Group	Dose (*μ*g·ml^−1^)	OD value	IR_1_ (%)
NS	—	0.2212 ± 0.0976	0
DMSO	—	0.1907 ± 0.0575	0
HY②	100	0.0760 ± 0.0357^*∗∗*^	60.1
	20	0.1233 ± 0.0221^*∗*^	35.3
	4	0.1860 ± 0.0305	2.5
HY③	100	0.1855 ± 0.0479	2.7
	20	0.1968 ± 0.0520	0
	4	0.1820 ± 0.0455	4.6
HY④	100	0.2043 ± 0.0733	0
	20	0.1867 ± 0.0477	2.1
	4	0.2100 ± 0.0753	0
HY⑤	100	0.2190 ± 0.0959	1.0
	20	0.2030 ± 0.0870	8.2
	4	0.2342 ± 0.1037	0
HY⑥	100	0.2258 ± 0.1024	0
	20	0.2142 ± 0.0973	3.2
	4	0.2273 ± 0.1093	0
NS	—	0.2243 ± 0.1047	0
DMSO	—	0.1908 ± 0.0519	0
HG②	100	0.0657 ± 0.0283^*∗∗*^	65.6
	20	0.1192 ± 0.0248^*∗*^	37.5
	4	0.1723 ± 0.0369	9.7
HG③	100	0.1942 ± 0.0584	0
	20	0.1893 ± 0.0678	0.8
	4	0.1917 ± 0.0668	0
HG④	100	0.1907 ± 0.0613	0.05
	20	0.2018 ± 0.0587	0
	4	0.2028 ± 0.0572	0
HG⑤	100	0.2281 ± 0.0981	0
	20	0.2040 ± 0.0867	9.1
	4	0.2268 ± 0.0825	0
HG⑥	100	0.2230 ± 0.0952	0.6
	20	0.2270 ± 0.0811	0
	4	0.2218 ± 0.0684	1.1

Note: compared with the DMSO control group, ^*∗*^*P* < 0.05, ^*∗∗*^*P* < 0.01.

**Table 2:** Effect of HY② and HG② on HuH-7 cells (*n* = 3, Mean ± SD).

Group	Dose (*μ*g·ml^−1^)	First experiment			Second experiment		
		OD value	IR_1_ (%)	IC_50_ (*μ*g·ml^−1^)	OD value	IR_1_ (%)	IC_50_ (*μ*g·ml^−1^)
PBS	—	0.1860 ± 0.0094	0	—	0.1914 ± 0.0161	0	—

DMSO	—	0.1753 ± 0.0081	0	—	0.1843 ± 0.0060	0	—

HG②	200	0.0307 ± 0.0142	82.5	46.58	0.0343 ± 0.0137	81.4	46.99
	100	0.0610 ± 0.0210	65.2		0.0653 ± 0.0215	64.6	
	50	0.0767 ± 0.0181	56.2		0.0827 ± 0.0188	55.1	
	25	0.1143 ± 0.0234	34.8		0.1193 ± 0.0199	35.3	
	12.5	0.1415 ± 0.0218	19.3		0.1468 ± 0.0190	20.3	

HY②	200	0.0409 ± 0.0121	76.7	58.92	0.0427 ± 0.0132	76.8	58.88
	100	0.0681 ± 0.0288	61.2		0.0706 ± 0.0287	61.7	
	50	0.0785 ± 0.0584	55.2		0.0817 ± 0.0574	55.7	
	25	0.1148 ± 0.0588	34.5		0.1184 ± 0.0617	35.7	
	12.5	0.1567 ± 0.0310	10.6		0.1509 ± 0.0305	9.5	

Epirubicin	20	0.0263 ± 0.0132	85.7	4.60	0.0281 ± 0.0097	84.9	4.79
	10	0.0653 ± 0.0150	64.5		0.0683 ± 0.0200	63.3	
	5	0.0907 ± 0.0232	50.7		0.0811 ± 0.0205	48.8	
	2.5	0.1127 ± 0.0101	38.7		0.1143 ± 0.0106	38.5	
	1.25	0.1503 ± 0.0166	18.2		0.1527 ± 0.0168	17.9	

**Table 3:** Correlation between common peaks of the active site and antitumor activity.

Peak	Correlation	Peak	Correlation	Peak	Correlation
1	0.666	13	0.836	25	0.664
2	0.608	14	0.781	26	0.796
3	0.395	15	0.656	27	0.656
4	0.888	16	0.728	28	0.539
5	0.696	17	0.863	29	0.854
6	0.731	18	0.666	30	0.878
7	0.896	19	0.549	31	0.431
8	0.780	20	0.549	32	0.855
9	0.574	21	0.844	33	0.762
10	0.449	22	0.341	34	0.538
11	0.734	23	0.511	35	0.988
12	0.847	24	1.000		

**Table 4:** Effect of medicated serum on tumor cell proliferation (*n* = 6, Mean ± SD).

Group	Medicated serum concentration (%)	OD value	IR_1_ (%)
Control group	0	0.1707 ± 0.0173	0

ip. group	50	0.0968 ± 0.0192^*∗*^	43.3
	25	0.1201 ± 0.0303^*∗*^	29.6
	12.5	0.1409 ± 0.0334	17.4

ig. group	50	0.1650 ± 0.0424	3.3
	25	0.1652 ± 0.0402	3.2
	12.5	0.1662 ± 0.0382	2.6

Note: compared with the control group, ^*∗*^*P* < 0.05.

**Table 5:** Analysis of tumor weight (*n* = 10, Mean ± SD).

Group	Administration routes	Dose (mg·kg^−1^)	Tumor weight (g)	IR_2_ (%)
Control group	ip + ig	—	1.582 ± 0.6581	—
FU group	Ip	83	0.340 ± 0.2212^*∗∗∗*^	78.51
ip. group	Ip	60	0.562 ± 0.3120^*∗∗*^	64.47
		20	1.058 ± 0.6898	33.12
ig. group	Ig	300	0.737 ± 0.5543^*∗*^	53.41
		100	1.078 ± 0.9428	31.86

Note: compared with the control group, ^*∗*^*P* < 0.05, ^*∗∗*^*P* < 0.01, and ^*∗∗∗*^*P* < 0.001.

**Table 6:** Pathological changes of tumor tissue and expression of the PCNA and VEGF (*n* = 10, Mean ± SD).

Group	Dose (mg·kg^−1^)	Scores^a^	PCNA^b^	VEGF^c^
Control group	—	4.20 ± 0.47	62.50 ± 3.06	167.96 ± 1.7662
FU group	83	6.50 ± 0.31^*∗*^	45.20 ± 4.30^*∗*^	146.15 ± 0.9609^*∗∗*^
ip. group	60	5.50 ± 0.37^*∗*^	50.00 ± 3.04^*∗*^	134.90 ± 0.5266^*∗∗*^
	20	4.70 ± 0.26	56.20 ± 4.05	134.87 ± 0.7009^*∗∗*^
ig. group	300	5.90 ± 0.35^*∗*^	47.80 ± 3.17^*∗*^	126.80 ± 2.4520^*∗∗*^
	100	4.70 ± 0.26	38.80 ± 2.95	141.043 ± 1.3292^*∗∗*^

Note: ^a^tumor cell lesions score (HE staining). ^b^Number of PCNA-positive tumor cells. ^c^Number of neovascularization in tumor tissue. ^d^Number of VEGF-positive tumor cells. Compared with the control group, ^*∗*^*P* < 0.05 and ^*∗∗∗*^*P* < 0.001.

## Data Availability

The data used to support the findings of this study are available from the corresponding author upon request.
